# Schizophrenia spectrum disorders in a Nigerian family: 4 case reports

**DOI:** 10.1186/1757-1626-2-14

**Published:** 2009-01-06

**Authors:** Joyce O Omoaregba, Bawo O James, George O Eze

**Affiliations:** 1 Department of Clinical Services, Federal Psychiatric Hospital, Uselu, P.M.B 1108, Benin City, Nigeria

## Abstract

**Introduction:**

The risk of developing schizophrenia is higher among persons with an affected sibling compared to the general population. Beliefs about aetiology influence presentation, management and treatment outcomes. There are no reports on multiple occurrences of this disorder in a family in this part of the world. In this case series we also highlight how cultural beliefs hamper and shape management.

**Case presentation:**

We report a case series of schizophrenia spectrum disorders affecting four women in a Nigerian family of Ibo tribal origin who responded marginally to pharmacological interventions and required insight oriented psychotherapy and family therapy in resolving psychosocial problems.

**Conclusion:**

While western taught disease models might explain these presentations, it is not usually accepted by lay persons from developing cultures. Limitations in contemporary treatment approaches necessitate the development of culturally relevant psychotherapeutic interventions.

## Introduction

Schizophrenia is believed to arise from a combination of genetic and environmental factors. With the observation that siblings of a proband have an 8.5% greater risk of developing the disorder compared to the general population [1], it is believed that the disorder may be of a polygenic multifactorial aetiology. It is has been estimated that about 70% of the variation in the liability to develop schizophrenia is accounted for by genes, with the remaining 30% of the variation being explained by the environment [2].

In developing countries, beliefs about the causation of mental illness among patients, caregivers and lay persons are at variation with established Western models of disease aetiology (which emphasize genetic susceptibility, environmental stresses and psychopathological constructs). Magico-religious reasons (possession by spirits, witchcraft, retribution and divine punishment) are believed by lay persons to cause mental illness [3-5]. Rosen argues that the 'externalizing beliefs' among lay persons in developing societies that mental illness may be caused by divine punishment, witchcraft or possession by spirits may partly be responsible for better treatment outcomes among the mentally ill. The patient is not seen as 'defective' or 'irredeemable' and community support during the phase of treatment and recovery shifts the focus away from the patient and alleviates caregiver burden [6]. These beliefs however, may hamper treatment offered by mental health professionals with a western styled education that focuses majorly on the patient (medication adherence, patient centred psychotherapeutic interventions) [7], rather than on the interrelationships between culture (environment) and physiology (genetics).

We present a case series of four sisters, three with ICD-10 diagnoses of schizophrenia, and one with a persistent delusional disorder. We highlight difficulties experienced in management, due to the influence of culture on beliefs about aetiology of the illness. A review of literature did not find similar presentations from Africa or other developing nations of the world.

## Case presentation

### Case 1

Miss P is a 49-year old woman, who is single and unemployed. She was first diagnosed with a psychiatric disorder at the age of 23. Six months prior to presentation, she stopped taking her prescribed medications, believing them to be poisoned and had become disruptive and talkative.

Over the next three months her sleep deteriorated, would often laugh aloud for no apparent reason and respond to questions asked her by relatives with irrelevant or incomprehensible answers. She often remained indoors, would not interact with family members and would become agitated when her privacy was intruded upon. She believed she was the 'wife of Jesus the Christ', and that she had been instructed through visions to 'keep herself pure and remain in isolation till Christ comes'. She refused meals prepared by her mother believing them to be poisoned.

On mental status examination, she was dishevelled and spoke in a high toned voice. Her speech was largely irrelevant and her thought content comprised themes of religion, possessing supernatural powers and of being persecuted by witches.

### Case 2

Mrs Q is a 48-year old married woman, who was having increasing difficulty co-ordinating house chores and caring for her children. She believed despite evidence to the contrary that her husband was unfaithful in their marriage.

She had suffered an acute psychotic breakdown following her first pregnancy when she was aged 28. She also suffered another relapse six years later, though the precipitating factor was not known. She had received care on in-patient basis on both occasions at another psychiatric facility in the country.

During interview, her husband complained that for over eight years, her belief about his unfaithfulness had led to embarrassing situations in which she had physically attacked relatives, clients or church members accusing them of either conniving with her husband to destroy her marriage or of being his sexual partner. She was often extravagant with finances and had several arguments with employees in a business she poorly ran.

She was kempt though irritable, unhappy and did not co-operate during the interview insisting she was not ill.

### Case 3

Miss X is a 42-year old single woman, who presented with complaints of talkativeness, poor sleep and wandering away from home. When prevented from leaving the home she often became physically aggressive and destroyed household property.

She would often weep uncontrollably, believing that her continued stay at home would result in her premature death. At other times she would withdraw to her room, refuse meals and would not talk to anyone. Her hygiene had deteriorated and this was an increasing burden on her caregiver.

On mental state examination, she had a depressed affect, with hesitant speech. She admitted to hearing voices that discussed her actions, ridiculed her and at times warned her not to interact with family members. Physical examination revealed a mean sustained blood pressure reading of 170/90 mmHg taking at two sittings five minutes apart.

### Case 4

Mrs Y is a 40-year old woman, who is separated from her husband and unemployed.

Y explained that whenever she had tried working, co-workers she would help financially, often became jealous and would plot to harm or kill her. She could not explain or provide evidence of how these persons tried to harm her. She admitted to hearing voices that advise her 'to fulfil her calling as a priestess,' these voices also discuss among themselves saying 'she would be a great person only if she obeys the call'. Over the last three years has been 'consulting' as a priestess for influential persons in society, however she has also had repeated conflicts with these clients over their refusal to obey her instructions.

She was unable to care for her children, and would leave them alone in her home for several days while she went about her 'job' as a priestess. She was agitated during the interview, her attention and concentration was poor, and she lacked insight.

#### Family structure

Four sisters: P, Q, X and Y, hail from a monogamous family of Ibo tribal origin. The father is deceased. There was no known history of mental illness in both parents and extended family members. Their developmental milestones and childhood history were uneventful.

There are eight siblings, six are women, the fourth and sixth are men. The eldest, a woman is deceased and had a history of mental illness. The third daughter is unaffected, and with her brothers provides financial support towards the care of three of their sisters (P, X, Y) who live with their mother. Q lives with her husband and children who bear the burden of her care.

#### Diagnoses and treatment

They (P, Q, X and Y)presented over a six month period to the Psychiatric Hospital, Benin City, Nigeria and were managed under one consultant psychiatrist. P became ill twenty two years ago which was about a year after the death of their father. Q became ill two years later after childbirth, while X and Y first became ill eleven years after the onset of illness in P. Haematological (full blood count, erythrocyte sedimentation rate) and biochemistry investigations (electrolytes and urea, blood sugar, urinalysis) workup were normal for all patients

#### Case 1

Following a careful review of her past history, a diagnosis of paranoid schizophrenia was made. She was commenced on clozapine, (maintained at 450 mg/day) following unsuccessful trials with trifluoperazine and risperidone at optimum doses over an 18 week period. There was a remarkable improvement in her mental state, which was evidenced by improvement in her personal hygiene and better social interaction, her delusions though less rigidly held persisted.

#### Case 2

She was managed for a persistent delusional disorder and over a thirteen week period was treated with risperidone 4 mg/day. She had several sessions of insight oriented psychotherapy and family therapy. Relations with her husband improved marginally

#### Case 3

A diagnosis of a schizoaffective disorder (depressed type) with essential hypertension as a co-morbidity was made. She responded well to Haloperidol 15 mg/day and Amitryptiline 100 mg/day, with a course of electroconvulsive therapy. Her blood pressure was effectively controlled with tablets nifedipine and a thiazide diuretic.

#### Case 4

She was managed as a case of paranoid schizophrenia, and was treated with tablets trifluoperazine 20 mg/day. Though she agreed that she erred in judgement regarding the care of her children, treatment and insight oriented psychotherapy did not alter her beliefs.

P, Q and X responded marginally to pharmacotherapy. While social interaction and personal hygiene improved over the course of treatment, they still held on to some delusional beliefs. These beliefs were often reinforced following visits by their mother. During family therapy, a suggestion that Y's practice as a priestess may not be the reason for the worsening of illness in P, Q, and X was met with resistance from Q, X and their mother. Their mother insisted that Y would not be allowed to return home to live with them if she continued to practice as a priestess. Her presence at home, it was believed attracted evil spirits to harm them during the incantations she had to recite when consulting or praying for her clients. The treatment team (psychiatrists, clinical psychologist and social workers) while not reinforcing this belief, had to explain to Y, that while not alluding to claims by other members of her family, living separately might help the outcome of her illness and that of her siblings. This form of therapy by manipulating the environment [8] was welcomed by Y, who believed that it will enable her obtain a paid job, practise her work as a diviner and save money to care for her children.

## Discussion

Though family studies provide a weak evidence base for genetic studies on schizophrenia, the pattern of presentation in this family is of interest. Inheritance in schizophrenia does not follow classical Mendelian patterns and a possible x-link paternally transmitted gene comes closest to explaining a genetic model for these presentations [9]. Information concerning the family history of these women was obtained from their mother. There may have been problems with recalling information; also she did not have much detail on the family history of her late husband.

Beliefs in supernatural causes for mental illness are widespread in Africa [3,5] and are even present in western societies [10]. Though those that hold such beliefs are sometimes in the minority [4], some of these beliefs promote stigma and discrimination [5]. Certain aspects of these beliefs are useful in therapy and cannot be ignored [6]. In this case series, it can be argued that the beliefs held by the patients and caregiver though not beneficial, required exploration, which was vital to treatment outcome.

Interventions by mental health professionals like in the case described are often empirical due to a paucity of research in the area of integrating culture in managing mental illness in this environment. Lack of a clear evidence base often deter specialists from instituting such interventions due to fears over criticism from peers and the potential for abusing such interventions. Involving family members in treatment, externalizing symptoms, employing a bio-psycho-socio-cultural-spiritual paradigm [6] are approaches that have been beneficial in this case series.

## Conclusion

Cultural influences on the presentation and outcome of mental illness require modification of treatment strategies to suit cases of mental illness especially in developing countries. There is a need to develop these culturally relevant treatment modalities to benefit the minority of psychiatric patients for whom cultural beliefs play a significant role in disease presentation, treatment and outcome.

## Consent

Written informed consent was obtained from the patients for publication of this case series. A copy of the written consent is available for review by the Editor-in-Chief of this journal.

## Competing interests

The authors declare that they have no competing interests.

## Authors' contributions

JOO, BOJ and GOE were involved in the direct management all four patients. JOO and BOJ conceived the idea for the manuscript. JOO, BOJ and GOE participated in the sequencing and drafting of the manuscript. All authors read and approved the final manuscript.
